# Inhibition of STAT3 alleviates LPS-induced apoptosis and inflammation in renal tubular epithelial cells by transcriptionally down-regulating TASL

**DOI:** 10.1186/s40001-023-01610-9

**Published:** 2024-01-06

**Authors:** Jin-Wen Xu, Ming-Yan Wang, Yan Mao, Zheng-Yun Hu, Xiao-Lin Miao, Feng Jiang, Guo-Ping Zhou

**Affiliations:** 1 Department of Pediatrics, The First Affiliated Hospital, Nanjing Medical University, Nanjing, China; 2 Department of Pediatric Nephrology, Wuxi Children’s Hospital Affiliated to Jiangnan University, Wuxi, China; 3 Department of Pediatrics, The Affiliated Hospital of Jiangsu University, Zhenjiang, China; 4 Department of Pediatrics, Songjiang Hospital Affiliated to Shanghai Jiao Tong University School of Medicine (Preparatory Stage), Shanghai, China; 5 Department of Neonatology, Obstetrics and Gynecology Hospital of Fudan University, Shanghai, China

**Keywords:** TASL, STAT3, Promoter, SLE

## Abstract

**Background:**

Systemic lupus erythematosus (SLE) is a common autoimmune disease that impacts various organs. Lupus nephritis (LN) significantly contributes to death in children with SLE. Toll-like receptor (TLR) adaptor interacting with SLC15A4 on the lysosome (TASL) acts as an innate immune adaptor for TLR and is implicated in the pathogenesis of SLE. A transcription factor known as signal transducer and activator of transcription 3 (STAT3), which is known to be linked to autoimmune diseases, is also involved in the development of SLE.

**Methods:**

Bioinformatics and real-time quantitative PCR (qRT-PCR) was used to detect the expression of STAT3 and TASL in peripheral blood of SLE patients and their correlation. Bioinformatics analysis, qRT-PCR, luciferase assay and chromatin immunoprecipitation (ChIP) were used to verify the regulation of transcription factor STAT3 on TASL. The expression levels of STAT3, TASL and apoptosis-related genes in LPS-induced HK2 cells were detected by qRT-PCR and Western blot. TUNEL staining were used to detect the apoptosis of HK2 cells after LPS stimulation. ELISA and qRT-PCR were used to detect the levels of inflammatory cytokines in the cell culture supernatant. TASL knockdown in HK2 cells was used to detect the changes in apoptosis-related genes and inflammatory factors. The expression level of TASL in LPS-stimulated HK2 cells and its effect on cell apoptosis and inflammatory factors were observed by knocking down and overexpressing STAT3, respectively. It was also verified in a rescue experiment.

**Results:**

The expressions of STAT3 and TASL were higher in SLE than in healthy children, and the expression of STAT3 was positively correlated with TASL. Transcription factor STAT3 can directly and positively regulate the expression of TASL through the promoter region binding site. The expression of STAT3, TASL and inflammatory cytokines was elevated, and the change of apoptosis was up-regulated in LPS-stimulated HK2 cells. Inhibition of STAT3 alleviates LPS-stimulated apoptosis and inflammatory response in HK2 cells through transcriptional regulation of TASL.

**Conclusions:**

These findings provide new insights into the transcriptional regulation of TASL and provide new evidence of a direct regulatory relationship between signaling nodes in the lupus signaling network.

**Graphical Abstract:**

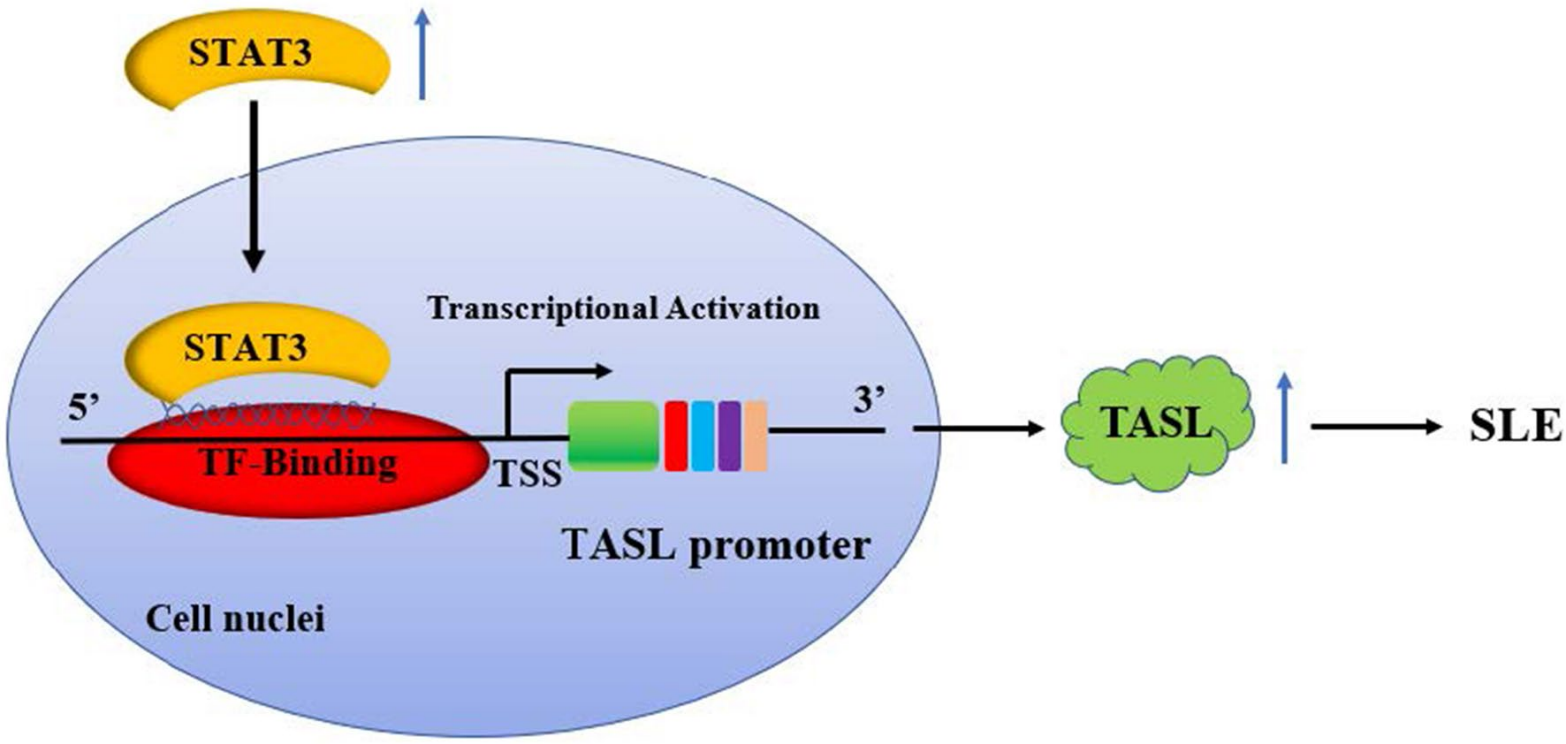

**Supplementary Information:**

The online version contains supplementary material available at 10.1186/s40001-023-01610-9.

## Introduction

Systemic lupus erythematosus (SLE) is a common autoimmune condition characterized by a systemic inflammatory response leading to the impairment of multiple systems and organs [1]. Despite SLE predominating in women, there has been a rise in cases among children in the past few years [2]. The most serious and common complication of SLE is lupus nephritis (LN), and it is responsible for causing the highest number of fatalities among children diagnosed with SLE [3]. The presence of autoantibodies and pro-inflammatory cytokines in lupus can develop LN, as they attack the kidneys and lead to renal dysfunction [4]. Currently, the primary treatment approach for SLE and LN involves using immunosuppressive drugs and glucocorticoids [5]. However, the prognosis of LN continues to be unfavorable because the cause of pathogenesis is not yet entirely understood. Treatment options for LN have been optimized in recent years, but the rate of complete renal response at 12 months is only 10–40% [6], and the incidence of end-stage renal disease after diagnosis is as high as 10.1% [7, 8].

The diagnostic criteria of pediatric SLE are the same as those of adults, and there are some similarities in clinical symptoms and immunological manifestations between pediatric SLE and adult SLE. The incidence of SLE in children has increased in recent years. Compared with adult-onset SLE, pediatric SLE is more aggressive, due to the variety of onset forms in children with SLE, the complex and severe clinical manifestations make the diagnosis of children with SLE difficult, and eventually the damage of important organs leads to poor prognosis. Therefore, it is imperative to identify novel treatment approaches to improve treatment outcomes.

Existing research has indicated that type I interferon (IFN) is significantly elevated in SLE [9]. Interferon alpha (IFN-α) is an IFN typically involved in viral defense and can potentially activate antigen-presenting cells after the uptake of their substances [10]. This process can ultimately lead to disruption of self-tolerance, making it a fundamental mechanism in the development of SLE [11]. Circulating levels of IFN-α are high in patients with SLE, and this high interferon phenotype is inheritable in SLE-affected families with a complex or polygenic inheritance pattern [12]. It has been shown that high serum IFN-α level is a genetic risk factor for the progression of SLE. Furthermore, pro-inflammatory factor production by monocytes/macrophages and IFN production by dendritic cells can trigger and exacerbate inflammation locally and systematically in individuals with SLE [13]. Signal transducer and activator of transcription (STAT) 4, interferon regulatory factor 5 (IRF5), IRF7, toll-like receptor (TLR) 7, and TLR9 are among the factors that may be involved in this process [14].

The X-linked gene Cxorf21 encodes the protein product TLR adaptor interacting with SLC15A4 on the lysosome (TASL), an immune adaptor protein for endolysosomal TLR7, TLR8, and TLR9 signaling transduction [14]. The pLxIS motif in TASL is critically involved in the recruitment and phosphorylation of IRF5 [15]. This event is closely linked to the progression of SLE [15]. In SLE, DNA, and RNA enter the cells of the intrinsic immune system as immune complexes, inducing TLR7, TLR8, and TLR9 responses in the endosomes [16]. This pathway leads to the production of interferons and other pro-inflammatory factors. TASL impacts the IRF5 pathway but not the NF-κB or MAPK signaling pathways in regulating gene expression, indicating a distinct function for TASL in activating natural immunity through endosomal TLRs [15].

The JAK/STAT pathway modulates various intracellular signal transduction processes, including cell proliferation, differentiation, and apoptosis [17]. One of its key components is STAT3. STAT3 is located on chromosome 12 (q13–q14-1), with a DNA length of 4815 bp and 24 exons, and encodes a protein (molecular weight of 84–113 kDa) with transcription factor activity [18]. STAT3 is downstream of and activated by many growth factors, cytokines, and chemokines [19]. Upon binding the ligand to the receptor, the receptor dimerizes and activates JAK, facilitating the phosphorylation and activation of the STAT3 protein [20]. Activated STAT3 protein forms heterodimer or homodimer, which then enters the nucleus to play a transcriptional role [21]. Prior evidence suggests that STAT3 is critically involved in SLE and that silencing STAT3 specifically in T cells can impede its capacity to assist B cells in producing autoantibodies and the induction of cellular infiltration into the tissue, which ultimately reduces kidney injury [22].

Herein, this research examines the function of the TASL gene in LN, a common complication caused by SLE. This research illustrated for the first time that transcriptional regulation of the TASL gene by STAT3 affects apoptosis and inflammation in LPS-induced HK2 cells. This study provides new ideas in molecular biology for developing targeted therapies against SLE-associated LN.

## Methods

### Bioinformatics analysis

The gene expression microarrays of individuals with SLE and normal healthy individuals were retrieved and then normalized by resequencing microarray (RMA). The differentially expressed genes (DEGs) were screened using the online tool GEO2R. Using |log_2_fold change (FC)|≥ 1 and adj. *P* < 0.05 as screening criteria, DEGs were obtained between patients with SLE and normal healthy individuals. The volcano plot was obtained using the R language package ggplot2. Twenty DEGs were selected, and the heatmap was plotted using GraphPad 9.0. To conduct a more detailed analysis of the identified DEGs, Gene ontology (GO) and Kyoto Encyclopedia of Genes and Genomes (KEGG) enrichment analyses were executed using the tool Metascape (https://metascape.org/). Protein–protein interaction (PPI) network studies were generated for DEGs utilizing STRING (https://cn.string-db.org).

### Blood sample collection

Peripheral blood of 6 LN patients and 5 healthy controls were collected. Ethylenediaminetetraacetic acid (EDTA) anticoagulation tubes were used to collect 2 mL whole blood samples from pediatric subjects with SLE and healthy pediatric subjects. The mononuclear cells were immediately extracted for RNA and detected for TASL and STAT3 expression. The approval for study protocols was granted by the Institutional Review Board of the Wuxi Children's Hospital Affiliated to Jiangnan University (WXCH2021-12-004). In addition, all the legal guardians of included children signed written informed consents before sample collection.

### Cell culture and model establishment

Human renal tubular epithelial cell line human kidney 2 (HK2) was purchased from the Cell Bank of the Chinese Academy of Sciences (Shanghai, China) and maintained in an incubator at 37 °C with 5% CO_2_. The cells were cultured in DMEM/F12 medium containing 10% fetal bovine serum and 1% antibiotic (penicillin/streptomycin). To simulate inflammation during LN model establishment, we treated HK2 cells with lipopolysaccharide (LPS) using the method described in the literature of Xu et al., to evaluate the effect produced by TASL during inflammation [23]. The same volume of phosphate-buffered saline (PBS) solution was used as a control.

### Cell transfection

Plasmid transfection was performed according to the instructions provided by Lipofectamine™ 3000 (Thermo Fisher). HEK293T and HK2 cells were seeded in well plates and cultured using penicillin and streptomycin-free medium. Transfection was performed when the cells grew to approximately 70–80% confluence. RNA samples were extracted after 24 h, and protein samples were extracted after 48 h.

### Quantification of luciferase activity

A dual-luciferase reporter gene assay kit (Promega, USA) was employed for the detection of luciferase activity. The STAT3 overexpression plasmid and small interfering RNA (siRNA) targeting STAT3 (siSTAT3) were co-transfected with the recombinant plasmid (Jinbeijin Biotechnology Co., Ltd., China) containing the TASL promoter fragment of luciferase and the internal reference plasmid (pRL-TK, Promega, USA), respectively. Luciferase activity was then assayed as per the provided guidelines. The firefly luciferase activity was normalized to Renilla luciferase activity.

### Quantitative real-time PCR (qRT-PCR)

The RNA-Quick Purification Kit (YISHAN Biotechnology., LTD, China) was utilized to extract total RNA, and PrimeScript RT Master Mix (TaKaRa, Japan) was employed for the synthesis of cDNA. The SYBR Green RT-PCR kit (Vazyme, China) was used to conduct fluorescence qRT-PCR assays. Relative gene expression was identified using the 2^−ΔΔCt^ method and normalized to GAPDH. Primers for TASL, STAT3, BCL-2, BAX, TNF-α, and IL6 were designed by TSINGKE (Beijing, China).

### Chromatin immunoprecipitation (ChIP)

The ChIP kit (Millipore, USA) was used for this assay, and DNA was purified and precipitated as per the provided guidelines for subsequent PCR analysis. PCR products were detected by electrophoresis on agarose gels. The SYBR kit (Vazyme, China) was employed to perform quantitative PCR of ChIP DNA.

### Cell viability assay

The cell viability was determined by means of the cell counting kit-8 (CCK-8) (MCE, USA) as per the provided guidelines. Optical density (OD) values at 450 nm were quantified using a microplate reader (Thermo Fisher, USA), followed by a viability analysis.

### Annexin V-FITC/PI apoptosis assay

The Annexin V-FITC/ PI staining kit (Vazyme, China) was used, and the tested cells were processed per the provided guidelines. In addition, a flow cytometer (Beckman Coulter, USA) was utilized in order to conduct an apoptosis assay.

### TUNEL staining

Using a fixation solution of 4% paraformaldehyde, the cells in the well plates were fixed for a duration of 30 min, following a 5-min treatment with 0.3% Triton X-100 at room temperature. Next, cells were subjected to incubation with TUNEL reagent (Beyotime, China) for 60 min. Finally, fluorescence microscopy was employed for the observation of cells sealed with a sealing agent containing DAPI.

### Enzyme-linked immunosorbent assay (ELISA)

Levels of inflammatory factors IL6 and TNF-α in cell supernatants were quantified with ELISA kits and then detected using a fully automated biochemical analyzer ADVIA® 2400 (Siemens, China).

### Western blot

After lysis buffer-based extraction of total proteins, equal amounts of protein extracts were separated by sodium dodecyl sulfate–polyacrylamide gel electrophoresis and transferred to polyvinylidene difluoride membranes. Afterward, 5% bovine serum albumin (BSA) was used as a blocking agent for 1 h at room temperature. The blots were probed with primary antibodies overnight at 4 °C. A horseradish peroxidase-conjugated secondary antibody (diluted at 1:5000) was subsequently introduced. Enhanced chemiluminescence reagents (Thermo Fisher Scientific, USA) were employed to visualize the protein bands. Primary antibodies, including anti-STAT3, anti-TASL, anti-cleaved caspase-3, anti-BAX, anti-Cleaved-Caspase3 and anti-BCL-2 were purchased from Abcam. Anti-β-actin antibody (loading control) and goat anti-rabbit IgG H&L (HRP) (secondary antibody) were purchased from Beyotime.

### Statistical analysis

The experimental results were presented as mean ± standard deviation, and GraphPad prism 9.0 software was employed for statistical analysis. The data between two groups were compared using an unpaired *t*-test, and *P* < 0.05 was considered statistically significantly different.

## Results

### TASL and STAT3 are correlated in SLE

**Fig. 1:**
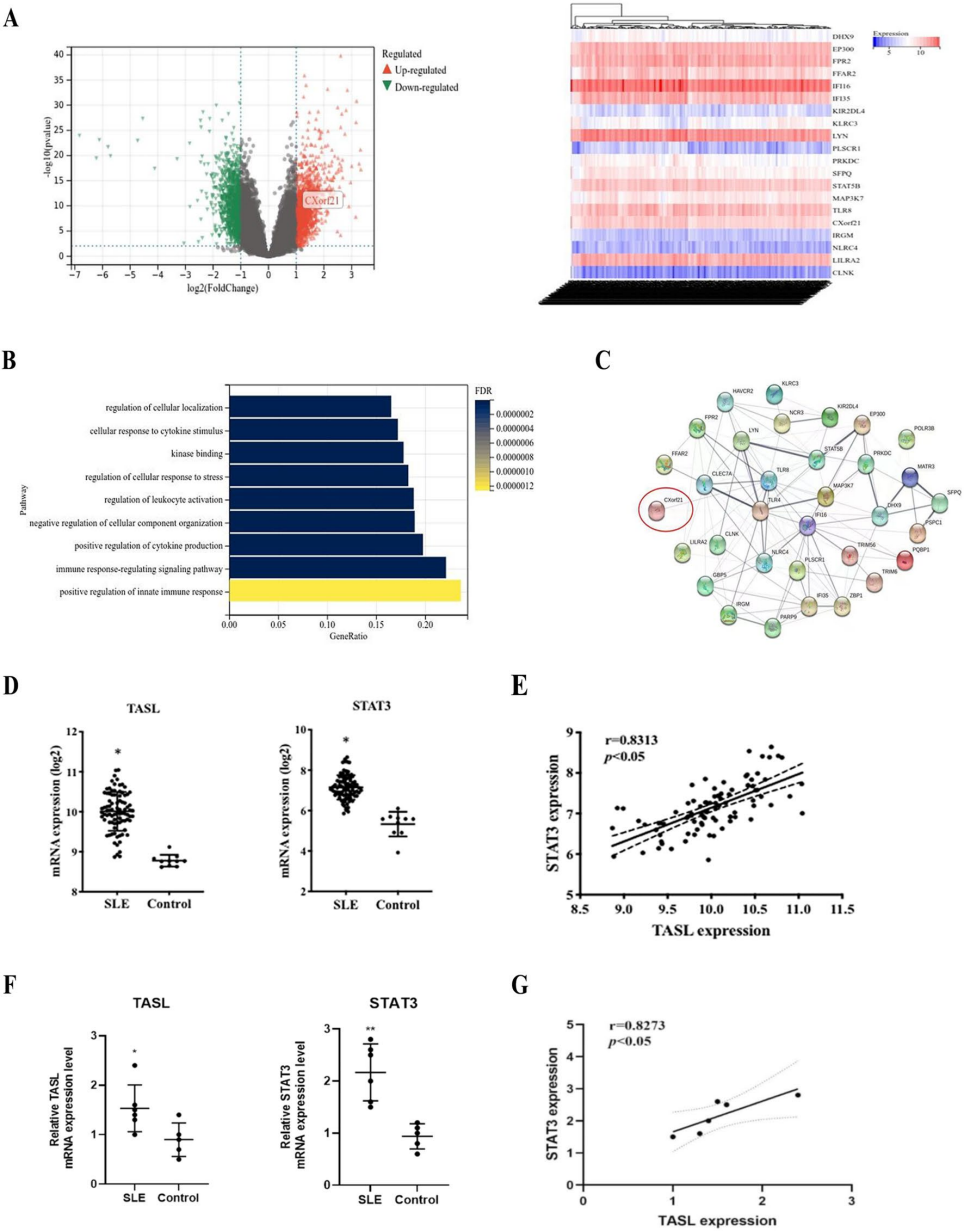
Bioinformatics analysis and clinical sample-based validation of the possible involvement of TASL with STAT3 in SLE. **A**, **B** GEO2R (**A**), GO and KEGG (**B**) analysis of the dataset; **C** PPI network constructed based on DEGs using the STRING database; **D**, **E** Expression (**D**) and correlation (**E**) analysis of TASL and STAT3 genes in the dataset; **F**, **G** TASL and STAT3 expression was detected (**F**) and correlation analysis was performed (**G**) based on the RNA extracted from PBMCs of healthy children and children with SLE (*P < 0.05, **P < 0.01)

In this study, the study subjects of the GSE185047 dataset were retrieved, and the microarray sequencing results were normalized. A total of 2620 DEGs, including 1301 down-regulated genes and 1319 up-regulated genes, were analyzed using the GEO2R tool. Among them, 20 DEGs were found to be significantly differentially expressed in peripheral blood mononuclear cells (PBMCs) of adult individuals with SLE compared with healthy individuals (Fig. 1A). The GO and KEGG analyses highlighted that DEGs were mainly enriched in such pathways as “intrinsic immune response, cytokines, immune response regulatory signaling pathway, leukocyte activation regulation, stress response regulation, kinase binding protein regulation” and other pathways (Fig. 1B). To further search for key nodes of DEGs, the genes were uploaded to the STRING online database to construct the PPI network. The results showed that the PPI network contained 32 node molecules, and TLR4, TLR8, and IFI16 were the core nodes of the PPI network, including the TLR8 molecular adaptor protein CXorf21 (Fig. 1C). The function of CXorf21 in SLE pathogenesis has been under investigation for several years, but the role of its protein product remained unclear until Heinz et al. discovered it to be an adaptor protein in TLR signaling contributing to the progression of SLE in 2020 [15]. Then, the gene was given a new name, TASL [15]. By analyzing the GSE185047 dataset, it was found that the expression of STAT3 and TASL was notably elevated in individuals with SLE than in healthy controls (Fig. 1D). STAT3 expression is critically involved in SLE and is associated with multi-organ damage [24]. Additional analysis indicated a strong positive relationship between the expression of STAT3 and TASL (Fig. 1E). Subsequently, the detection of RNA expression in PBMCs in healthy and diseased pediatric subjects highlighted that the mRNA expression of TASL and STAT3 was considerably elevated in children with SLE than in healthy children (Fig. 1F). The Pearson correlation analysis revealed a positive relationship between the expression of STAT3 and TASL in children with SLE (Fig. 1G). These results highlight that TASL triggers the development of SLE, and its gene expression is correlated with STAT3.

### TASL gene is positively regulated by the transcription of STAT3

**Fig. 2:**
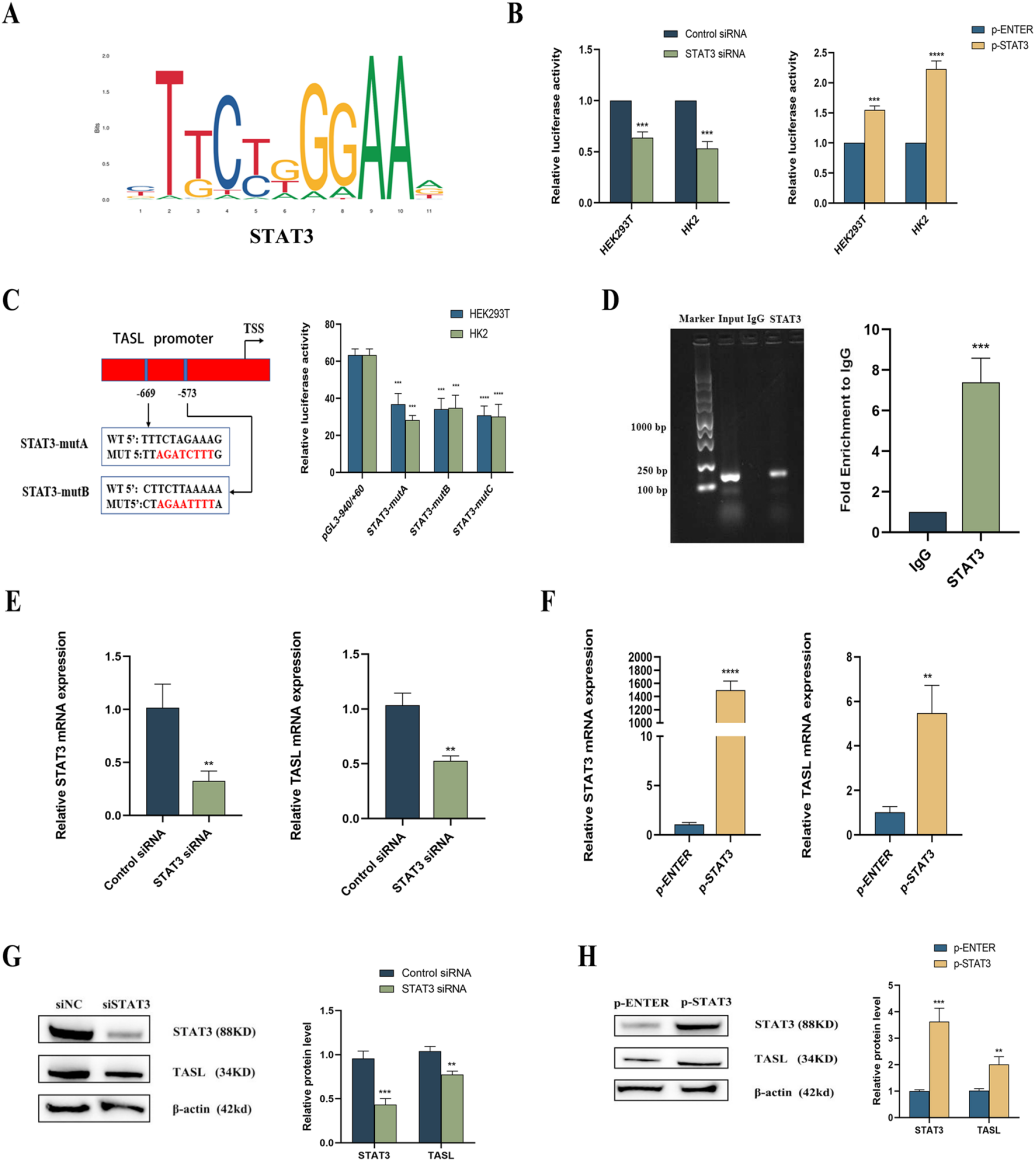
Bioinformatic prediction and experimental confirmation of the regulatory relationship between STAT3 and TASL. **A** STAT3 binding motif map in the TASL promoter region predicted by JASPAR; **B** detection of luciferase activity in HEK293T and HK2 cells after co-transfection of siSTAT3 and pSTAT3 with luciferase recombinant plasmid containing TASL gene promoter, respectively; **C** construction of the point mutant plasmid of two STAT3 binding sites in the TASL promoter region and determination of luciferase activity after transfection; **D** ChIP-qPCR and agarose gel electrophoresis assay for STAT3 binding in the TASL promoter region; **E**, **F** qRT-PCR for mRNA expression of STAT3 and TASL after transfection with siSTAT3 (**E**) and pSTAT3 (**F**); **G**, **H** western blot assay to detect the protein levels of STAT3 and TASL after transfection with siSTAT3 **G** and pSTAT3 **H** (**P < 0.01, ***P < 0.001, ****P < 0.0001)

The application of the JASPAR website predicted the presence of two STAT3 binding sites in the region from − 940 bp to + 60 bp of the TASL promoter (Fig. 2A and Additional file 1: Fig. S1A). The TASL gene promoter from − 940 bp to + 60 bp was cloned into the pGL3-Basic luciferase reporter gene plasmid (Additional file 1: Fig. S1B), and the product of the recombinant plasmid digested by double digestion with KpnI and NheI restriction endonucleases was subsequently subjected to agarose gel electrophoresis. The results showed that two specific bands appeared at around 4.8 kb and 1 kb, indicating that the TASL promoter fragment-containing pGL3-Basic plasmid was successfully constructed (Additional file 1: Fig. S1C). The constructed pGL3-Basic vector containing the TASL promoter fragment and the negative control (NC) pGL3-Basic plasmid were transfected into HEK293T and HK2 cells. The luciferase reporter gene assay showed that the luciferase activity of the gene with the TASL promoter fragment was considerably higher compared with the NC pGL3-Basic (Additional file 1: Fig. S1D), indicating that the region from − 940 bp to + 60 bp of the TASL gene is a functional promoter. The TASL promoter-containing gene plasmid, along with siSTAT3, control siRNA, pSTAT3 or pENTER were transfected into HEK293T cells and HK2 cells, respectively. The results indicated that the TASL gene promoter activity was markedly reduced in the siSTAT3 group when compared with the control siRNA, while the overexpression of STAT3 in the pSTAT3 group increased the human TASL gene promoter activity (Fig. 2B). Subsequently, point mutant plasmids were constructed separately for the two binding sites of the human TASL gene with STAT3, named STAT3-mutant (mut)A and STAT3-mutB. Additionally, simultaneous mutations were introduced to STAT3-mutA and STAT3-mutB, resulting in a mutant named STAT3-mutC. Compared with the original plasmid, promoter activities were decreased by 48.6% after the mutation of STAT3-mutA alone and by 45.5% after the mutation of STAT3-mutB alone. It was decreased by 51.7% when both sites were mutated simultaneously, with no further significant decrease compared to single-point mutation (Fig. 2C). To further clarify the binding of transcription factor STAT3 to TASL promoter, ChIP-qPCR and agarose gel electrophoresis in HK2 cells indicated that the binding of STAT3 to TASL promoter region was notably elevated than that of non-specific IgG control (Fig. 2D). To investigate the impact of STAT3 on TASL mRNA and protein expression, siSTAT3 and pSTAT3 were separately transfected into HK2 cells. qRT-PCR and Western blot findings highlighted that TASL mRNA and protein levels were considerably decreased in the siSTAT3 group compared with the NC group, while they were remarkably elevated in the pSTAT3 group (Fig. 2E–H).

### Involvement of TASL in LPS-induced injury to HK2 cells

**Fig. 3:**
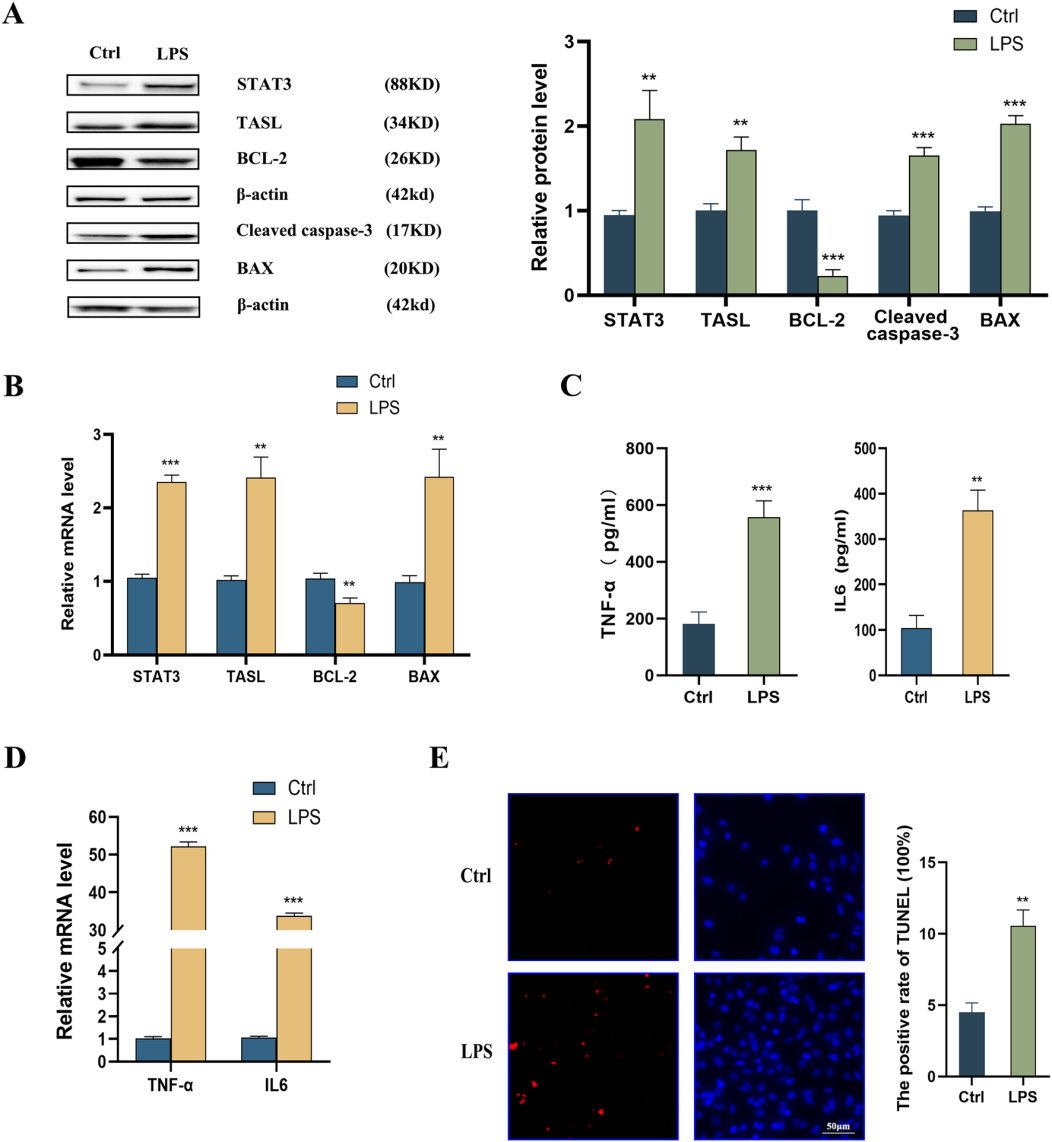
Effect of LPS induction on inflammation and apoptosis of HK2 cells. **A** Western blot to detect the protein levels of STAT3, TASL, BCL-2, cleaved caspase-3, and BAX in LPS-induced HK2 cells; **B** qRT-PCR to detect the mRNA expression of STAT3, TASL, BCL-2 and BAX in LPS-induced HK2 cells; **C** ELISA to detect the inflammatory factors IL6 and TNF-α levels in the supernatant of LPS-induced HK2 cells; **D** qRT-PCR to detect mRNA expression of IL6 and TNF-α in LPS-induced HK2 cells; **E** TUNEL staining to detect apoptosis in LPS-induced HK2 cells (**P < 0.01, ***P < 0.001)

Multiple studies have adopted LPS-induced HK2 cells to create an in vitro LN cell model [23]. Cell viability was measured after LPS stimulation of HK2 cells, and the results showed that cell viability was elevated after 10 μg/mL LPS treatment for 24 h (Additional file 1: Fig. S1E). Western blot assay highlighted that LPS induced a considerable decrease in BCL-2 levels and increased protein levels of cleaved caspase-3, BAX, STAT3, and TASL in HK2 cells (Fig. 3A). Meanwhile, qRT-PCR assay revealed that mRNA expression of BAX, STAT3, and TASL was elevated, and that of BCL-2 was decreased (Fig. 3B). In addition, ELISA highlighted that the inflammatory factors TNF-α and IL6 levels were notably enhanced in the supernatant of LPS-treated HK2 cells (Fig. 3C), while the mRNA expression of TNF-α and IL6 was also enhanced simultaneously (Fig. 3D). TUNEL-based analysis indicated that LPS treatment resulted in a considerable increase in apoptotic cells (Fig. 3E). In conclusion, the expression of STAT3 and TASL was elevated, and cell apoptosis and inflammation were also increased in the LPS-induced in vitro LN model.

### Knockdown of TASL expression attenuates LPS-induced apoptosis and inflammation in HK2 cells

**Fig. 4:**
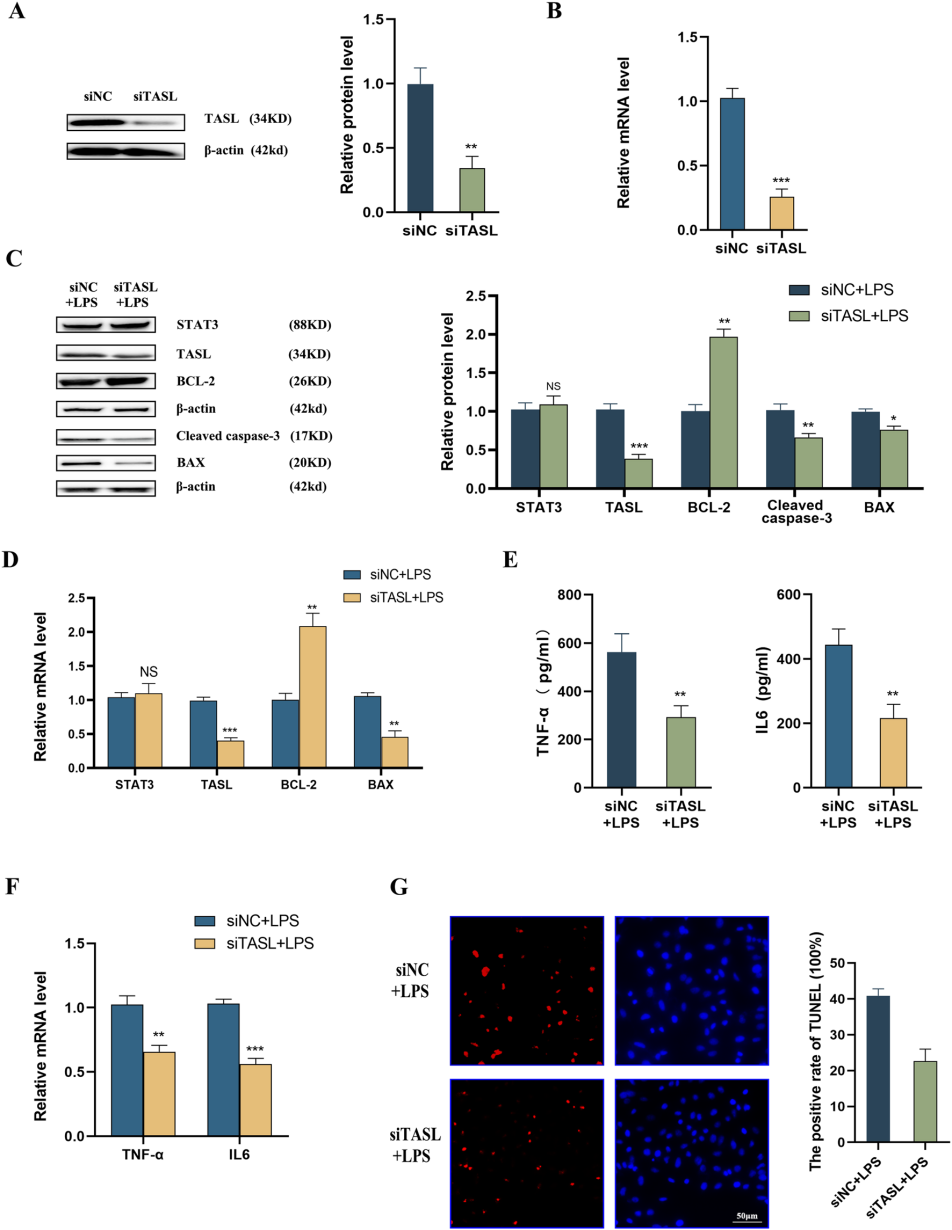
Effect of silencing of TASL on LPS-induced inflammation and apoptosis in HK2 cells. **A**, **B**, Protein (**A**) and mRNA (**B**) expression of TASL in HK2 cells after transfection with siTASL; **C** western blot to detect the protein levels of STAT3, TASL, BCL-2, cleaved caspase-3, and BAX in LPS-induced HK2 cells after silencing of TASL; **D** qRT-PCR to detect the mRNA expression of STAT3, TASL, BCL-2, and BAX in LPS-induced HK2 cells after silencing of TASL; **E** ELISA to detect the levels of inflammatory factors IL6 and TNF-α in the supernatants of LPS-induced HK2 cells after silencing of TASL; **F** qRT-PCR to detect the mRNA expression of inflammatory factors IL6 and TNF-α in the supernatants of LPS-induced HK2 cells after silencing of TASL; **G** TUNEL staining to detect apoptosis of in LPS-induced HK2 cells after silencing of TASL (*P < 0.05, **P < 0.01, ***P < 0.001)

After transfection of siTASL into HK2 cells, mRNA and protein levels of TASL were considerably reduced (Fig. 4A, B). Silencing of TASL in LPS-induced HK2 cells significantly down-regulated protein levels of cleaved caspase-3 and BAX and up-regulated BCL-2 levels, while STAT3 levels were not significantly changed (Fig. 4C). Meanwhile, qRT-PCR results showed decreased mRNA expression of BAX and increased expression of BCL-2 (Fig. 4D). ELISA indicated that the inflammatory factors IL6 and TNF-α were considerably reduced in the supernatant of LPS-challenged HK2 cells after the knockdown of TASL expression (Fig. 4E). Consistent results were shown by qRT-PCR, where the mRNA levels of TNF-α and IL6 were also reduced (Fig. 4F). In addition, it was observed that LPS treatment notably elevated the percentage of apoptotic cells in TUNEL staining, and silencing of TASL expression counteracted this up-regulation (Fig. 4G). Thus, TASL is involved in LPS-induced renal tubular epithelial cell injury, and inhibition of its expression could curb apoptosis and inflammation.

### Involvement of STAT3 in LPS-induced injury in HK2 cells

**Fig. 5:**
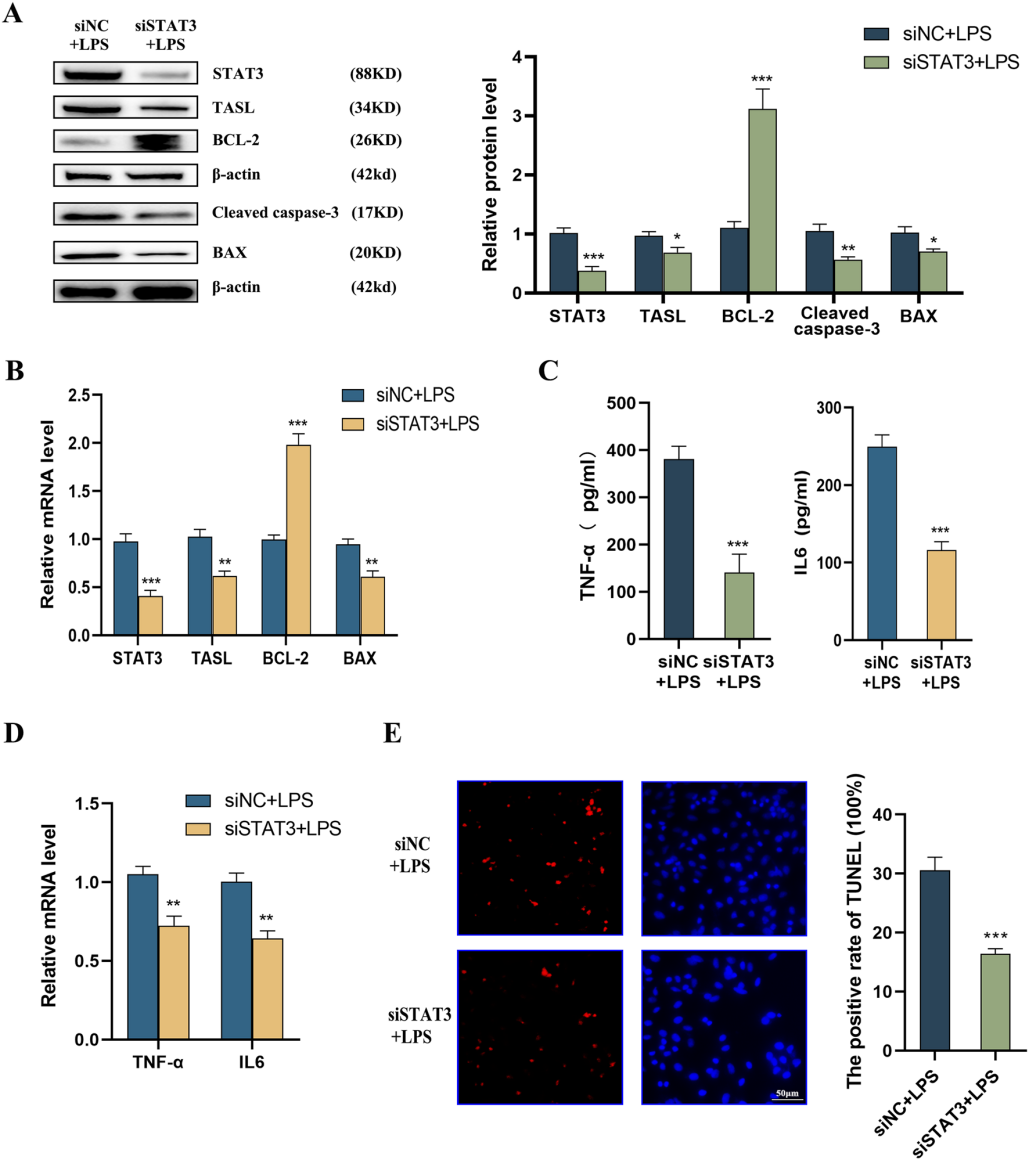
Effect of silencing of STAT3 on LPS-induced inflammation and apoptosis in HK2 cells. **A** Western blot to detect the protein levels of STAT3, TASL, cleaved caspase-3, BCL-2, and BAX in LPS-induced HK2 cells after silencing of STAT3; **B** qRT-PCR to detect the mRNA expression of STAT3, TASL, BCL-2, and BAX in LPS-induced HK2 cells after silencing of STAT3; **C** ELISA to detect the levels of inflammatory factors IL6 and TNF-α in the supernatants of LPS-induced HK2 cells after silencing of STAT3; **D** qRT-PCR to detect the mRNA expression of IL6 and TNF-α in LPS-induced HK2 cells after silencing of STAT3; **E** TUNEL staining to detect the apoptosis in LPS-induced HK2 cells after silencing of STAT3 (*P < 0.05, **P < 0.01, ***P < 0.001)

In LPS-induced HK2 cells, Western blot showed that knockdown of STAT3 down-regulated STAT3, TASL, cleaved caspase-3, and BAX protein levels, accompanied by an increase in BCL-2 protein levels (Fig. 5A). qRT-PCR highlighted that mRNA expression of STAT3, TASL, and BAX was notably reduced, accompanied by an increase in BCL-2 expression (Fig. 5B). ELISA showed that knockdown of STAT3 down-regulated the levels of inflammatory factors IL6 and TNF-α in the supernatants of LPS-induced HK2 cells (Fig. 5C). Their mRNA expression was also reduced, as evidenced by qRT-PCR assay (Fig. 5D). TUNEL staining showed that the knockdown of STAT3 in LPS-stimulated HK2 cells significantly reduced the percentage of apoptotic cells (Fig. 5E). The above results suggest that the knockdown of STAT3 can suppress apoptosis and inflammation in LPS-stimulated HK2 cells.

### TASL is transcriptionally regulated by STAT3 to affect LPS-induced injury in HK2 cells

**Fig. 6:**
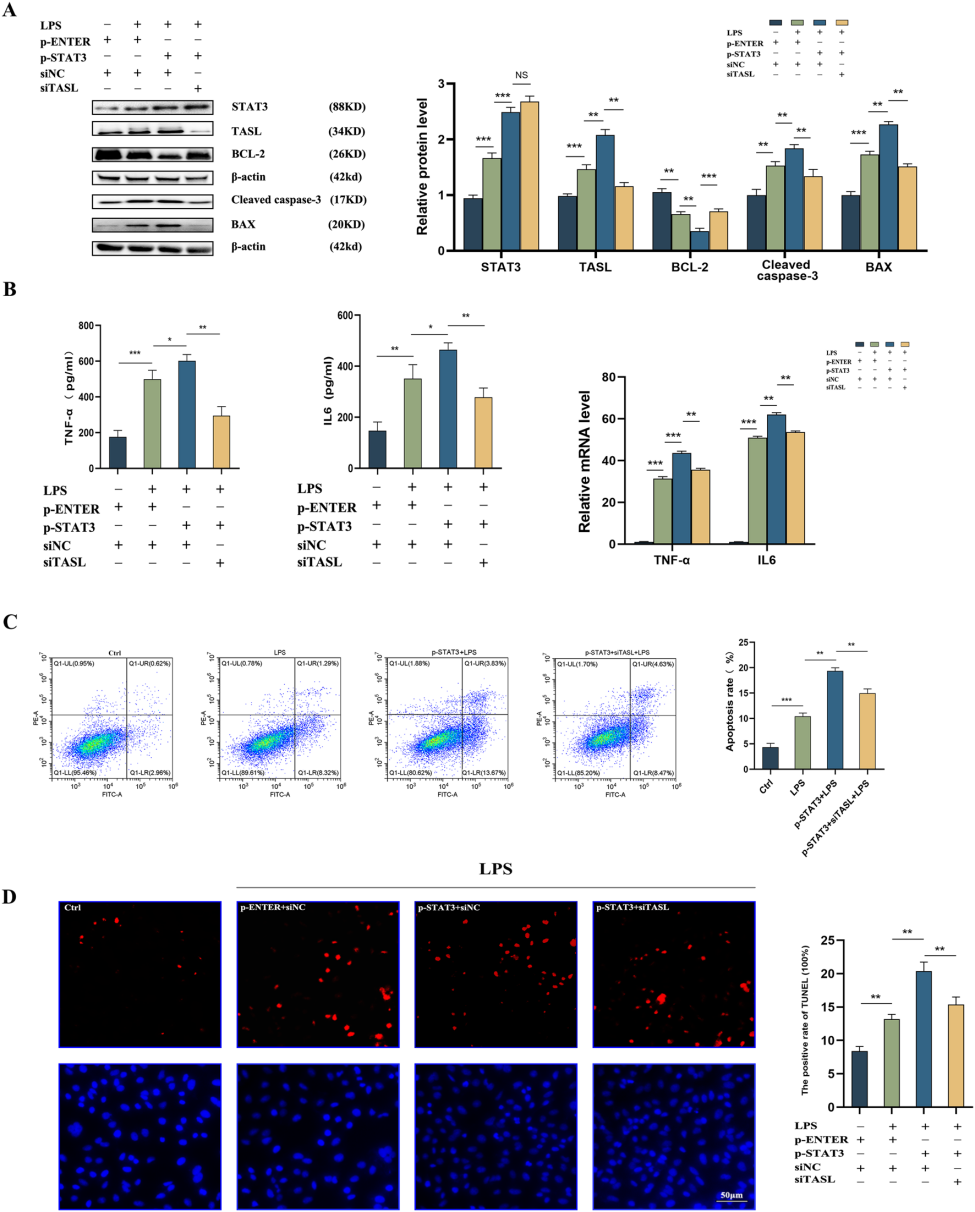
Effect of STAT3 overexpression and TASL silencing on LPS-induced inflammation and apoptosis in HK2 cells. **A** Western blot assay to detect the protein levels of STAT3, TASL, BCL-2, cleaved caspase-3, and BAX in LPS-induced HK2 cells in response to STAT3 overexpression and TASL silencing; **B** ELISA and qRT-PCR assay to detect the levels of IL6 and TNF-α in LPS-induced HK2 cells in response to STAT3 overexpression and TASL silencing. **C** Flow cytometry to detect apoptosis in LPS-induced HK2 cells in response to STAT3 overexpression and TASL silencing; **D** TUNEL staining to detect apoptosis in LPS-induced HK2 cells in response to STAT3 overexpression and TASL silencing (*P < 0.05, **P < 0.01, ***P < 0.001)

Functional rescue assays were performed to further investigate whether STAT3 affected apoptosis and inflammation through transcriptional regulation of TASL. Western blot results showed that the protein levels of STAT3, TASL, cleaved caspase-3, and BAX were elevated, while the BCL-2 protein level was reduced after ectopic expression of STAT3, indicating elevated cell apoptosis. In contrast, transfection of siTASL negated the elevated apoptosis caused by overexpression of STAT3 (Fig. 6A). Meanwhile, ELISA results indicated that inflammatory factors IL6 and TNF-α in the supernatants were notably increased in LPS-treated HK2 cells after overexpression of STAT3, while knockdown of TASL attenuated the worsening of inflammation. The qRT-PCR results found that in LPS-treated HK2 cells overexpressing STAT3, IL6, and TNF-α mRNA expression was down-regulated after silencing of TASL (Fig. 6B). In addition, flow cytometric results showed that boosted apoptosis was identified in LPS-treated HK2 cells overexpressing STAT3, while the apoptosis was curbed after the knockdown of TASL expression (Fig. 6C). Meanwhile, TUNEL staining showed that knockdown of TASL in LPS-induced HK2 cells overexpressing STAT3 significantly decreased the TUNEL-positive rate of the cells (Fig. 6D). Therefore, all the above results suggest that the transcriptional regulation of TASL by STAT3 affects apoptosis and inflammation in LPS-induced HK2 cells.

## Discussion

SLE is characterized by the excessive production of autoantibodies and immune complexes that attack self-nuclear antigens [25]. This leads to damage in several target organs. Activated immune cells eventually produce inflammatory cytokines, including TNF-α, IL6, and IFN-α. In SLE, LN significantly contributes to morbidity and mortality and poses a severe threat to organ function [26]. The development and progression of LN involve multiple factors, and its underlying causes and mechanisms are not entirely comprehended. Individuals with LN demonstrate elevated levels of autoantibodies in circulation, which combine with autoantigens to form systematic or in situ immune complexes [27]. The accumulation of immune complexes and complement components in the glomerular and tubular interstitium leads to the death of intrinsic renal cells [28]. It is accompanied by reduced clearance of dead cells, resulting in inflammatory damage to the kidney [29]. Programmed cell death releases many molecular markers associated with injury, such as adenosine triphosphate (ATP) and high mobility group protein 1 (HMG1), which attack and activate pro-inflammatory cells primarily through TLRs. Activation of inflammasomes in macrophages of patients with SLE and mice with SLE-like phenotypes enhances inflammation and autoimmunity, leading to LN episodes and organ damage [30, 31]. Pro-inflammatory cells trigger a positive feedback loop by producing cytokines such as TNF-α, promoting further cell death. The collective impact of these processes contributes to the progression of LN [32]. Available evidence suggests that cytokines such as TNF-α, IL6, and IFN-α are critically involved in the pathogenesis and disease activity of SLE by regulating immune functions, and that overexpression of these cytokines can lead to exacerbation of LN [33]. Although the administration of glucocorticoids or other immunosuppressive agents has enhanced the prognosis for individuals with LN, their usage is frequently associated with drug resistance and significant adverse effects related to treatment [34].

TASL is an X-linked gene associated with SLE that encodes a protein that interacts with SLC15A4 [15]. SLC15A4 belongs to the solute carrier family and is an amino acid transporter protein localized within the lysosome [35]. It regulates antigen processing in the lysosome and TLR7/9-mediated inflammatory responses in the endosome and triggers IFN-α production in dendritic cells involved in SLE pathogenesis [36]. The inclusion of TASL as an adaptor protein is a new and important finding that must be incorporated into the blueprint of regulatory interactions in lysosomal compartments and interactions between acidification and signaling through IRF5. Hydroxychloroquine is the first-line drug for the treatment of SLE. It is a weak base that accumulates in the lysosomal lumen and can reduce the acidity of lysosomes, thereby inhibiting various functions of lysosomes, including autophagy, Toll-like receptor activation in endosomes and calcium signaling pathway [37]. Another mechanism of action of hydroxychloroquine can block TLR7 and TLR9, the signaling of which leads to the production of interferon-α and its effector cytokines. Interferon-α is an important mediator in the pathogenesis of SLE, and increased expression of interferon-induced genes is found in most SLE patients [38].

Using bioinformatics tools, this research predicted that the transcription factor STAT3 could bind to the promoter of TASL and control the expression of the TASL gene. Further, it was demonstrated that TASL promoter activity could be regulated by STAT3 by dual-luciferase assay, transcription factor overexpression assay, and RNA interference assay. Through the ChIP assay, we demonstrated in vitro that STAT3 could bind to the TASL promoter region. By validation at the mRNA and protein levels, it was shown that STAT3 has a facilitative effect on TASL expression. This means that STAT3 can promote the transcription of the TASL gene by binding to the corresponding binding site in the promoter region of the TASL gene.

This research revealed the function of the TASL gene in an in vitro LN cell model and explored its possible mechanisms. Our work uncovered that knockdown of the TASL gene attenuated LPS-induced inflammatory injury in HK2 cells by reducing levels of inflammatory factors (IL6 and TNF-α) and suppressing apoptosis in HK2 cells. These findings shed light on possible molecular mechanisms for clinical implications and inspire a new therapeutic idea in LN treatment. Therefore, this study anticipates that TASL may be a promising target for gene therapeutics of LN. Due to the predominance of autoimmune females, the field has tended to view sex differences through a pathological lens, that is, autoimmunity, emphasizing how proteins like TASL contribute to the production of deleterious agents. Identification of the CXorf21 protein product as TASL is an important step in understanding male and female differences in SLE and other immune-mediated diseases, and a deeper understanding of the TASL protein provides new targets for the treatment of SLE.

To sum up, our work demonstrated that the transcription factor STAT3 can directly bind to the promoter region of TASL and up-regulate the promoter activity and mRNA and protein expression of TASL. Thus, it lays the foundation for studying the molecular mechanism of transcriptional regulation of the TASL gene and provides a promising target for gene therapeutics of SLE.

## Conclusions

In conclusion, our work indicates that STAT3 transcriptionally regulates TASL in SLE-induced LN to affect apoptosis and inflammation. Furthermore, the knockdown of TASL expression down-regulated apoptosis and inflammation in an in vitro LN model induced by LPS. These findings provide new insights into the transcriptional regulation of TASL and provide new evidence of a direct regulatory relationship between signaling nodes in the lupus signaling network.

### Supplementary Information

**Additional file 1: Figure S1.** Identification of the binding between TASL and STAT3.

## Data Availability

The datasets used and/or analyzed during the current study are available from the corresponding author on reasonable request.

## Abbreviations

SLE: Systemic lupus erythematosus
LN: Lupus nephritis
TASL: Toll-like receptor (TLR) adaptor interacting with SLC15A4 on the lysosome
STAT3: Signal transducer and activator of transcription 3
qRT-PCR: Quantitative real-time PCR
LPS: Lipopolysaccharide
IFN: Interferon
IFN-α: Interferon alpha
IRF5: Interferon regulatory factor 5

## References

[1] Lazar S, Kahlenberg JM (2023). Systemic lupus erythematosus: new diagnostic and therapeutic approaches. Annu Rev Med.

[2] Satterthwaite AB (2021). TLR7 signaling in lupus B cells: new insights into synergizing factors and downstream signals. Curr Rheumatol Rep.

[3] Yu C, Li P, Dang X (2022). Lupus nephritis: new progress in diagnosis and treatment. J Autoimmun.

[4] Chang A, Clark MR, Ko K (2021). Cellular aspects of the pathogenesis of lupus nephritis. Curr Opin Rheumatol.

[5] Chen XC, Wu D, Wu HL (2022). Metformin improves renal injury of MRL/lpr lupus-prone mice via the AMPK/STAT3 pathway. Lupus Sci Med..

[6] Almaani S, Meara A, Rovin BH (2017). Update on lupus nephritis. Clin J Am Soc Nephrol.

[7] Hanly JG, O'Keeffe AG, Su L (2016). The frequency and outcome of lupus nephritis: results from an international inception cohort study. Rheumatology.

[8] Pesce F, Stea ED, Rossini M (2020). Glomerulonephritis in AKI: from pathogenesis to therapeutic intervention. Front Med.

[9] Crow MK (2014). Advances in understanding the role of type I interferons in systemic lupus erythematosus. Curr Opin Rheumatol.

[10] Psarras A, Alase A, Antanaviciute A (2020). Functionally impaired plasmacytoid dendritic cells and non-haematopoietic sources of type I interferon characterize human autoimmunity. Nat Commun.

[11] Liu W, Zhang S, Wang J (2022). IFN-gamma, should not be ignored in SLE. Front Immunol.

[12] Niewold TB, Hua J, Lehman TJ (2007). High serum IFN-alpha activity is a heritable risk factor for systemic lupus erythematosus. Genes Immun.

[13] Sengupta S, Bhattacharya G, Mohanty S (2023). IL-21, inflammatory cytokines and hyperpolarized CD8(+) T cells are central players in lupus immune pathology. Antioxidants.

[14] Teruel M, Alarcon-Riquelme ME (2016). The genetic basis of systemic lupus erythematosus: what are the risk factors and what have we learned. J Autoimmun.

[15] Heinz LX, Lee J, Kapoor U (2020). TASL is the SLC15A4-associated adaptor for IRF5 activation by TLR7-9. Nature.

[16] Schwickert TA, Tagoh H, Schindler K (2019). Ikaros prevents autoimmunity by controlling anergy and Toll-like receptor signaling in B cells. Nat Immunol.

[17] Couronne L, Scourzic L, Pilati C (2013). STAT3 mutations identified in human hematologic neoplasms induce myeloid malignancies in a mouse bone marrow transplantation model. Haematologica.

[18] De Groof A, Ducreux J, Aleva F (2020). STAT3 phosphorylation mediates the stimulatory effects of interferon alpha on B cell differentiation and activation in SLE. Rheumatology.

[19] Zhong Z, Wen Z, Darnell JE (1994). Stat3: a STAT family member activated by tyrosine phosphorylation in response to epidermal growth factor and interleukin-6. Science.

[20] Forbes LR, Milner J, Haddad E (2016). Signal transducer and activator of transcription 3: a year in review. Curr Opin Hematol.

[21] Edwards LJ, Mizui M, Kyttaris V (2015). Signal transducer and activator of transcription (STAT) 3 inhibition delays the onset of lupus nephritis in MRL/lpr mice. Clin Immunol.

[22] Yoshida N, He F, Kyttaris VC (2019). T cell-specific STAT3 deficiency abrogates lupus nephritis. Lupus.

[23] Xu ZQ, Ding Y, Huang XY (2021). CircELK4 contributes to lupus nephritis by acting as a miR-27b-3p sponge to regulate STING/IRF3/IFN-I signaling. Inflammation.

[24] Rahman A, Isenberg DA (2008). Systemic lupus erythematosus. N Engl J Med.

[25] Cortini A, Ellinghaus U, Malik TH (2017). B cell OX40L supports T follicular helper cell development and contributes to SLE pathogenesis. Ann Rheum Dis.

[26] Liu BC, Tang TT, Lv LL (2018). Renal tubule injury: a driving force toward chronic kidney disease. Kidney Int.

[27] Azzouz D, Omarbekova A, Heguy A (2019). Lupus nephritis is linked to disease-activity associated expansions and immunity to a gut commensal. Ann Rheum Dis.

[28] Gomez Mendez LM, Cascino MD, Garg J (2018). Peripheral blood B cell depletion after rituximab and complete response in lupus nephritis. Clin J Am Soc Nephrol.

[29] Yung S, Chan TM (2017). Molecular and immunological basis of tubulo-interstitial injury in lupus nephritis: a comprehensive review. Clin Rev Allergy Immunol.

[30] Westerterp M, Gautier EL, Ganda A (2017). Cholesterol accumulation in dendritic cells links the inflammasome to acquired immunity. Cell Metab.

[31] Kahlenberg JM, Carmona-Rivera C, Smith CK (2013). Neutrophil extracellular trap-associated protein activation of the NLRP3 inflammasome is enhanced in lupus macrophages. J Immunol.

[32] Brightbill HD, Suto E, Blaquiere N (2018). NF-kappaB inducing kinase is a therapeutic target for systemic lupus erythematosus. Nat Commun.

[33] Adamichou C, Georgakis S, Bertsias G (2019). Cytokine targets in lupus nephritis: current and future prospects. Clin Immunol.

[34] Barber MRW, Clarke AE (2020). Systemic lupus erythematosus and risk of infection. Expert Rev Clin Immunol.

[35] Song F, Yi Y, Li C (2018). Regulation and biological role of the peptide/histidine transporter SLC15A3 in Toll-like receptor-mediated inflammatory responses in macrophage. Cell Death Dis.

[36] Harris VM, Harley ITW, Kurien BT (2019). Lysosomal pH is regulated in a sex dependent manner in immune cells expressing CXorf21. Front Immunol.

[37] Nirk EL, Reggiori F, Mauthe M (2020). Hydroxychloroquine in rheumatic autoimmune disorders and beyond. EMBO Mol Med.

[38] Lambers WM, Westra J, Bootsma H (2021). Hydroxychloroquine suppresses interferon-inducible genes and B cell activating factor in patients with incomplete and new-onset systemic lupus erythematosus. J Rheumatol.

