# Supplementary material for: Inhibition of STAT3 alleviates LPS-induced apoptosis and inflammation in renal tubular epithelial cells by transcriptionally down-regulating TASL
Source: Eur J Med Res. 2024 Jan 6;29:34. doi: 10.1186/s40001-023-01610-9 (PMC10770942; doi:10.1186/s40001-023-01610-9)
Supplement: Supplementary file 1 — Additional file 1: Figure S1. Identification of the binding between TASL and STAT3. [file 40001_2023_1610_MOESM1_ESM.docx]

**Figure S1 Identification of the binding between TASL and STAT3.**

**
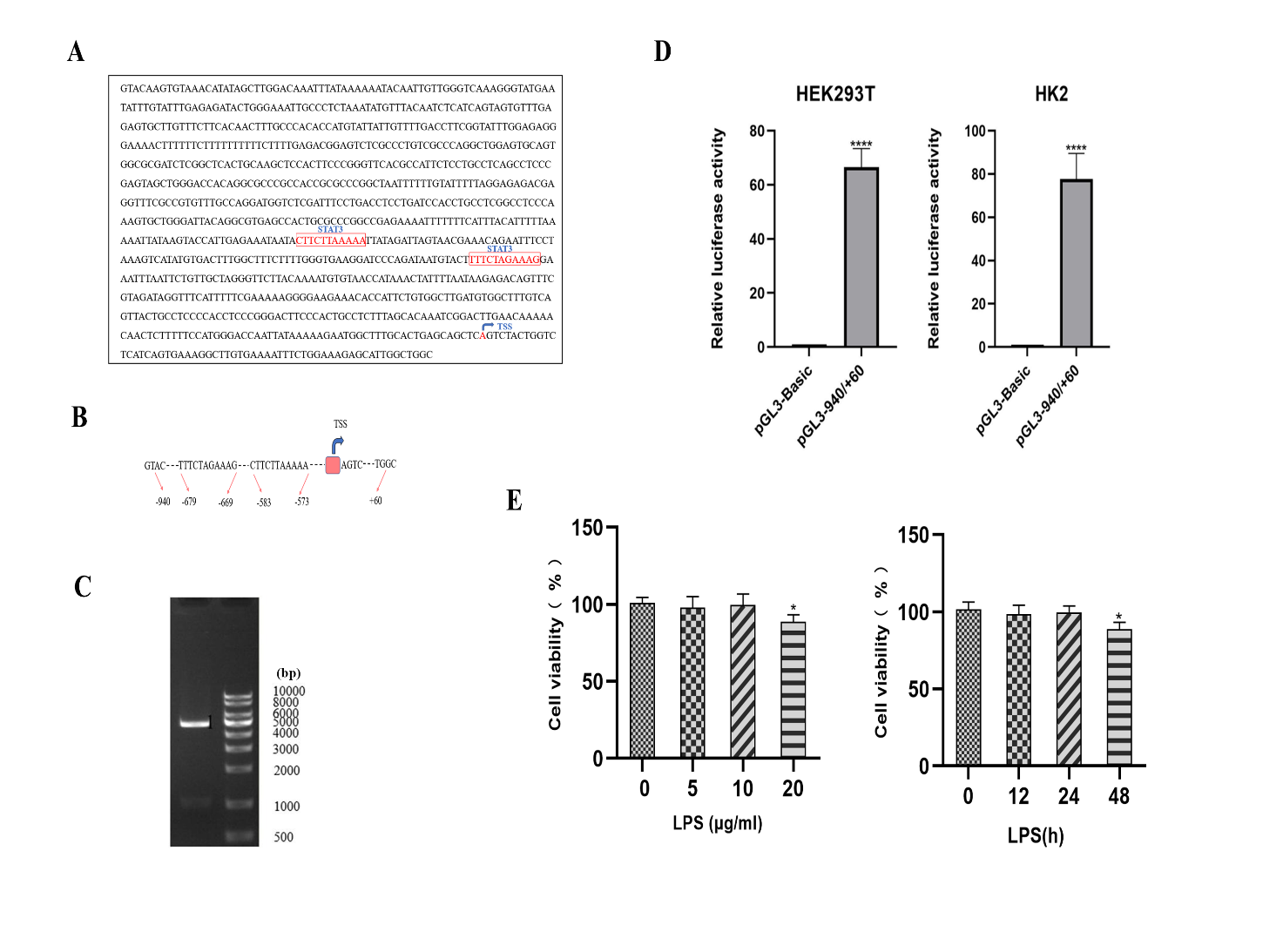
**

A, Binding site of STAT3 in the region from -940 bp to +60 bp of the TASL promoter;

B, Schematic diagram of the sequence of the promoter region of the TASL gene inserted into the pGL3-Basic plasmid;

C. Identification of enzyme digestion of recombinant reporter plasmids containing the sequence (from -940 bp to +60 bp) of the TASL gene promoter;

D, Detection of luciferase activity of HEK293T and HK2 cells transfected with the pGL3-Basic plasmid containing TASL promoter;

E, Cell viability detected by the CCK-8 kit in LPS-induced HK2 cells(* *P* < 0.05, *****P* < 0.0001).
